# Iron Deficiency Is Associated With Reduced Levels of *Plasmodium falciparum-specific* Antibodies in African Children

**DOI:** 10.1093/cid/ciaa728

**Published:** 2021-07-01

**Authors:** Caroline K. Bundi, Angela Nalwoga, Lawrence Lubyayi, John Muthii Muriuki, Reagan M. Mogire, Herbert Opi, Alexander J. Mentzer, Cleopatra K. Mugyenyi, Jedida Mwacharo, Emily L. Webb, Philip Bejon, Thomas N. Williams, Joseph K. Gikunju, James G. Beeson, Alison M. Elliott, Francis M. Ndungu, Sarah H. Atkinson

**Affiliations:** 1 Kenya Medical Research Institute (KEMRI) Centre for Geographic Medicine Coast, KEMRI-Wellcome Trust Research Programme, Kilifi, Kenya; 2 Department of Medical Laboratory Science, Jomo Kenyatta University of Agriculture and Technology, Nairobi, Kenya; 3 Medical Research Council/Uganda Virus Research Institute and London School of Hygiene & Tropical Medicine Uganda Research Unit, Entebbe, Uganda; 4 Burnet Institute, Melbourne, Australia; 5 Wellcome Centre for Human Genetics, Nuffield Department of Medicine, University of Oxford, Oxford, United Kingdom; 6 Big Data Institute, Li Ka Shing Centre for Health Information and Discovery, University of Oxford, Oxford, United Kingdom; 7 MRC Tropical Epidemiology Group, Department of Infectious Disease Epidemiology, London School of Hygiene & Tropical Medicine, London, United Kingdom; 8 Centre for Tropical Medicine and Global Health, Nuffield Department of Medicine, University of Oxford, Oxford, United Kingdom; 9 Department of Medicine, Imperial College, London, United Kingdom; 10 Department of Microbiology, and Central Clinical School, Monash University, Melbourne, Australia; 11 Department of Medicine, University of Melbourne, Victoria, Australia; 12 Department of Clinical Research, London School of Hygiene & Tropical Medicine, London, United Kingdom, and; 13 Department of Paediatrics, University of Oxford, Oxford, United Kingdom

**Keywords:** iron deficiency, immunity, children, malaria, Africa

## Abstract

**Background:**

Iron deficiency (ID) and malaria are common causes of ill-health and disability among children living in sub-Saharan Africa. Although iron is critical for the acquisition of humoral immunity, little is known about the effects of ID on antibody responses to *Plasmodium falciparum* malaria.

**Methods:**

The study included 1794 Kenyan and Ugandan children aged 0–7 years. We measured biomarkers of iron and inflammation, and antibodies to *P*. *falciparum* antigens including apical merozoite antigen 1 (anti-AMA-1) and merozoite surface antigen 1 (anti-MSP-1) in cross-sectional and longitudinal studies.

**Results:**

The overall prevalence of ID was 31%. ID was associated with lower anti-AMA-1 and anti-MSP-1 antibody levels in pooled analyses adjusted for age, sex, study site, inflammation, and *P. falciparum* parasitemia (adjusted mean difference on a log-transformed scale (β) −0.46; 95 confidence interval [CI], −.66, −.25 *P* < .0001; β −0.33; 95 CI, −.50, −.16 *P* < .0001, respectively). Additional covariates for malaria exposure index, previous malaria episodes, and time since last malaria episode were available for individual cohorts. Meta−analysis was used to allow for these adjustments giving β −0.34; −0.52, −0.16 for anti−AMA−1 antibodies and β −0.26; −0.41, −0.11 for anti-MSP-1 antibodies. Low transferrin saturation was similarly associated with reduced anti-AMA-1 antibody levels. Lower AMA-1 and MSP-1-specific antibody levels persisted over time in iron-deficient children.

**Conclusions:**

Reduced levels of *P*. *falciparum-specific* antibodies in iron-deficient children might reflect impaired acquisition of immunity to malaria and/or reduced malaria exposure. Strategies to prevent and treat ID may influence antibody responses to malaria for children living in sub-Saharan Africa.

Iron deficiency (ID) is highly prevalent among young children living in sub-Saharan Africa [1], and iron deficiency anemia (IDA) is the leading cause of years lived with disability among African children [2] due to its negative effects on child development [3]. Malaria is also a major public health problem causing approximately 405 000 deaths in 2018, of which 85% occurred in sub-Saharan Africa, mainly among young children [4]. Children acquire immunity to malaria over time and antibodies to merozoite antigens are important mediators of naturally-acquired immunity [5, 6], in addition to other responses.

Iron is important for the development of humoral immunity and antibody production. ID impairs B-cell proliferation and antibody production [7], and a mutation in transferrin receptor 1 (TfR1), which causes insufficient cellular iron uptake, leads to defective B- and T-cell activation and combined-immunodeficiency [8]. ID is associated with reduced antibody levels in children [9–11] and in rat models [12], as well as with weakened vaccine responses [7, 13], although other studies have found little association with antibody levels [14–16] or vaccine responses [17, 18]. ID has also been associated with reduced frequencies of B and T cells and cytokines, necessary for antibody production [8–10, 19].

Although ID is highly prevalent among African children and is known to influence immune responses little is known about the effect of ID on the acquisition of immunity to malaria. We previously observed that ID was associated with decreased total immunoglobulin G (IgG) and immunoglobulin E (IgE) levels to *P. falciparum* schizont extract [20] and that hepcidin, the master iron-hormone, was associated with increased levels of antibodies to anti-AMA-1 and anti-MSP-2 antigens [21], in small studies. In the current study, we investigated the relationship between iron status and antibody levels to specific *P*. *falciparum* antigens in 1794 Kenyan and Ugandan children. We evaluated antibodies to 2 major merozoite antigens, anti-AMA-1 and anti-MSP-1, which are targets of acquired immunity, and antibodies to these antigens have previously been associated with protective immunity to malaria in our study population [5, 22].

## Materials And Methods

### Ethical Approval

Ethical approval was provided by the Scientific Ethics Review Unit of the Kenya Medical Research Institute (KEMRI/SERU/ CGMR-C/046/3257/2983), by the Uganda Virus Research Institute (reference GC/127/12/07/32), the Uganda National Council for Science and Technology (MV625), and in the United Kingdom by the London School of Hygiene & Tropical Medicine Ethics Committee (A340) and the Oxford Tropical Research Ethics Committee (OXTREC, 39-12 and 42-14 and 37-15).

### Study Population

We used data from community-based cohorts of children in Kilifi, Kenya, and Entebbe, Uganda.

#### Kenya

The Kenyan children included two community-based cohorts exposed to varying levels of malaria transmission, Junju and RTS,S. Junju is a surveillance cohort evaluating immunity to malaria as described elsewhere [23]. The RTS,S cohort is an extension of the RTS,S/AS01E vaccine trial against malaria conducted between 2007 and 2008 [24]. Both cohorts are under active weekly surveillance to assess for fever, and a malaria blood film is taken if the temperature is > 37.5°C. Additionally, annual cross-sectional blood samples are taken for immunology and parasitology during the dry period before the main annual malaria transmission season. Iron biomarkers and malaria antibodies were measured on the same plasma sample from a single annual cross-sectional bleed based on the availability of a sample archived at −80°C.

#### Uganda

The Entebbe Mother and Baby Study (EMaBS) is a prospective birth cohort that was originally designed as a randomized double-blind placebo-controlled trial to determine whether anthelmintic treatment during pregnancy and early childhood was associated with differential responses to vaccination or incidence of infections such as malaria, pneumonia and diarrhea [25]. Children had active surveillance for malaria and other infections during fortnightly home visits and quarterly clinic visits, and an annual blood sample was collected. Malaria antibodies were measured from a sample taken at 5 years of age, and iron biomarkers were measured from a single annual bleed taken between 1 and 4 years of age based on the availability of plasma samples archived at −80°C.

### Laboratory Procedures

#### Iron and Inflammation Biomarkers

The measured biomarkers of iron status and inflammation were iron (MULTIGENT iron calorimetric assay, Abbott Architect, USA), ferritin, transferrin (chemiluminescent microparticle immunoassay [CMI], Abbott Architect), soluble transferrin receptor (sTfR, Human sTfR ELISA, BioVendor), hepcidin (DRG Hepcidin 25 [bioactive] high sensitive ELISA Kit, DRG Diagnostics), transferrin (CMI, Abbott Architect) hemoglobin (Coulter analyzer, Beckman Coulter), and C-reactive protein (CRP, MULTIGENT CRP Vario assay, Abbott Architect). In Uganda, hemoglobin concentrations were adjusted for an altitude of > 1000 m above sea level (by subtracting 0.2 g/dL) [26]. *P. falciparum* parasitemia was determined at the time of malaria antibody measurement using Giemsa-stained thick and thin blood smears.

#### Plasmodium falciparum Antibody Assays

Antibodies against the AMA1 3D7 sequence and MSP1_42_, 3D7 sequence of *P falciparum* antigens were measured from plasma samples by enzyme-linked immunosorbent assays (ELISAs) according to standard protocols as previously described for the RTS,S, Junju [27] and EMaBS cohorts [28]. A pool of malaria hyperimmune sera was serially diluted on each plate, and the optical densities from these dilutions were used to generate a standard curve. From this standard curve, an arbitrary unit per milliliter (AU/mL) was calculated for each sample based on the relative optical density obtained. Different pools of malariahyperimmune sera and ELISA antigens were used in the Kenyan and Ugandan laboratories.

### Definitions

Inflammation was defined as CRP > 5 mg/L. ID was defined as plasma ferritin < 12 μg/L or < 30 μg/L in the presence of inflammation in children < 5 years or < 15 μg/L in children ≥ 5 years as defined by the World Health Organization (WHO) [29]. Low transferrin saturation (TSAT) < 10% (calculated as iron in μmol/L/(transferrin in g/L × 25.1) × 100)) [30] was considered as a secondary definition of ID. TSAT was calculated in Kenya only because Ugandan plasma samples were stored in EDTA, which chelates iron. We did not define ID by hepcidin or sTfR since there are no internationally established cutoffs. Anemia was defined as hemoglobin < 11 g/dL in children aged 0-4 years or hemoglobin < 11.5 g/dL in children >4 years. IDA was defined as low ferritin and anemia [1]. We used malaria exposure index, which estimates a distance-weighted local prevalence of malaria infection within a kilometer radius around an index child with acute malaria, as previously described [31]. A malaria episode was defined as parasitemia and temperature > 37.5°C. Malaria incidence was calculated by dividing total malaria episodes by follow-up time. Underweight was defined as weight-for-age *z*-score < -2 using the WHO Growth Reference Standards [32]. Hemoglobin and anthropometric measurements were only available for the Junju and EMaBS cohorts.

### Statistical Methods

Data analysis was performed using STATA 13.0 (StataCorp., College Station, TX). Non-normally distributed iron and inflammation biomarkers, and malaria antibodies were normalized by natural-log transformation. Crude differences in means of log antibody levels between iron-deficient and iron-replete children were determined using 2-tailed Student *t*-tests and univariable linear regression models. Multivariable linear regression models were used to estimate the association between ID and malaria antibody levels, pooling data from all cohorts and adjusting for study site, age, sex, inflammation, and *P*. *falciparum* parasitemia at the time of antibody measurement. The linear regressions were run on log transformed data; hence the beta values returned reflect changes on a log scale. To transform these to fold differences that could be applied to the linear scale, we used the formula 10^Beta. The significance of possible interactions was estimated from the Wald test. Because not all indexes of previous malaria exposure were available for all cohorts, a meta-analysis of results from multivariable models for individual cohorts was fitted, additionally adjusting for malaria exposure index in the Kenyan cohorts, malaria vaccination status in the RTS,S cohort, and prior malaria incidence, time since last malaria episode, and time between iron and antibody measurements in the EMaBS cohort. In longitudinal analyses EMaBS children were further grouped by those that had iron measurements 0–2 or 2–4 years before antibody measurement. A *P*-value of < .05 was considered significant.

## Results

A total of 924 Kenyan and 870 Ugandan children were included in the study. Participant characteristics and malaria exposure varied by study cohorts as shown in Table 1. Children living in Junju had the highest malaria exposure, whereas children in the RTS,S cohort had very low levels of malaria exposure. Prevalence of asymptomatic *P*. *falciparum* parasitemia varied by study cohort from 34.70% in Junju to 1.97% in the RTS,S cohort (Table 1).

Overall, 31% of children had ID as defined by WHO guidance [29], 47% had ID as defined by TSAT < 10%, and 13% had IDA, with prevalences varying by study cohort. Geometric means of individual iron biomarkers similarly showed that ID was highly prevalent among the cohorts. Malaria-specific antibody levels also varied by cohort. In keeping with a higher exposure to malaria, children in the Junju cohort had the highest levels of anti-AMA-1 and MSP-1 antibodies compared to other cohorts (Table 1). In univariable analyses, age, inflammation and malaria parasitemia were positively associated with both anti-AMA-1 and anti-MSP-1 antibody levels (Supplementary Table 1).

### Iron Deficiency Is Associated With Reduced Malaria-specific Antibody Levels

Anti-AMA-1 and anti-MSP-1 antibodies were lower in iron-deficient compared to iron-replete children, with the largest differences in antibody levels observed in children from the Junju cohort, where malaria transmission was also the highest (Supplementary Figure 1). In multivariable regression models adjusted for age, sex, study site, inflammation, and malaria parasitemia ID was associated with decreased anti-AMA-1 and anti-MSP-1 antibody levels (adjusted mean difference on a log-transformed scale (β) −0.46; 95% CI, −.66, −.25 *P* < .0001; β −0.33; 95% CI, −.50, −.16 *P* < .0001, corresponding to ~4 fold and 2-fold reductions on a linear scale, respectively) (Table 2). ID remained associated with reduced malaria-specific antibody levels after further adjustment for underweight (Supplementary Table 2).

**Figure 1:**
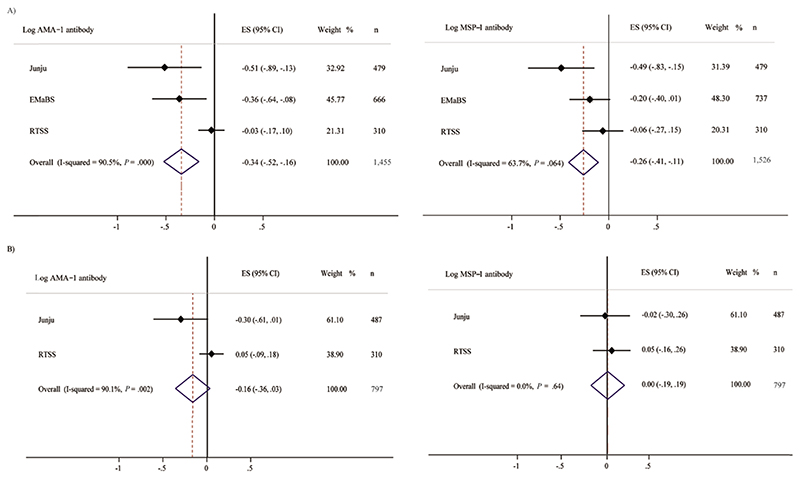
Meta-analyses of association of iron deficiency with AMA-1 and MSP-1 malaria antibodies. *A*, ID, low ferritin; *B*, ID, low TSAT. Regression models were adjusted for age, sex, inflammation, and malaria parasitemia in all individual cohorts. We additionally adjusted for malaria exposure index in Kenyan cohorts, malaria vaccination (RTS,S cohort), and malaria incidence, time since last malaria episode, and time between iron and antibody measurements (EMaBS cohort). ID was defined as plasma ferritin < 12 μg/L or < 30 μg/L in the presence of inflammation in children < 5 years or < 15 μg/L in children ≥ 5 years. Malaria exposure index, a marker of the level of a child’s exposure to malaria, was calculated as the distance-weighted prevalence of clinical malaria within 1 km radius of the child’s residence. Malaria incidence was defined as total number of malaria episodes/follow-up time. Abbreviations: AMA-1, apical merozoite protein 1; CI, confidence interval; EMaBS, Entebbe Mother and Baby Study; ES, effect size; ID, iron deficiency; MSP-1, merozoite surface protein 1.

To account more fully for the effects of previous malaria on antibody levels, we conducted a meta-analysis of individual cohorts with further adjustments for additional covariates including malaria exposure index [31], incidence of malaria prior to antibody measurement and time since last malaria episode, as available for individual cohorts. ID remained associated with decreased antibody levels to anti-AMA-1 (β −0.34; 95% CI, −.52, −.16) and anti-MSP-1 (β −0.26; 95% CI, −.41, −.11) in overall meta-analyses (Figure 1). IDA was associated with reduced anti-AMA-1 and anti-MSP-1 antibody levels in Kenyan children but not in Ugandan or in overall analyses (Supplementary Table 2 and Supplementary Figure 2).

Considering other iron biomarkers, we found that ID defined by TSAT < 10% was associated with reduced anti-AMA-1 but not anti-MSP-1 antibody levels in multivariable analyses and meta-analyses (Table 2 and Figure 1). Increased TSAT, ferritin, and sTfR levels were associated with increased anti-AMA-1 antibody levels, whereas higher ferritin and hepcidin levels were associated with increased anti-MSP-1 antibody levels in multivariable models (Supplementary Table 3).

### Iron Deficiency Earlier in Life May Influence Subsequent Antibody Levels

We tested the hypothesis that ID might influence subsequent malaria antibody levels for a prolonged period of time in the EMaBS birth cohort. ID was associated with lower anti-AMA-1 and anti-MSP-1 antibody levels up to 2 years after iron status measurements and only with lower anti-AMA-1 antibody levels 2–4 years after iron measurements (Supplementary Figure 3).

## Discussion

We have investigated the association between iron status and anti-*P*. *falciparum* antibodies in 1794 Kenyan and Ugandan children. We found that ID was associated with reduced levels of anti-AMA-1 and anti-MSP-1 antibodies, even after adjustment for potential confounders including previous malaria exposure. TSAT < 10% was similarly associated with reduced levels of anti-AMA-1 antibody levels. A range of individual iron markers, including ferritin, TSAT, hepcidin, and sTfR levels were positively associated with malaria antibody levels. ID remained associated with reduced malaria antibody levels for up to 4 years.

We found that ID was associated with lower anti-AMA-1 and anti-MSP-1 malaria antibody levels, even after adjustment for potential confounders including previous malaria exposure. The relationship between malaria, iron parameters, and antibody levels differed between study sites. The effect of ID on anti-*P*. *falciparum* antibody levels was most marked among children with the highest malaria exposure and antibody levels, as seen in the Junju cohort, although little difference by ID was observed in children with very low levels of malaria exposure and antibody levels, as seen in the RTS,S cohort. In agreement with our findings, Nyakeriga et al reported that total IgG, IgG2, and IgE antibody levels were lower in iron-deficient compared to iron-replete Kenyan children [20]. We found that the effects of ID on anti-AMA-1 and anti-MSP-1 antibody levels persisted over time, perhaps due to continuing ID or a long-term effect of ID on immune development. IDA was similarly associated with reduced anti-AMA-1 and anti-MSP-1 antibody levels in Kenyan but not Ugandan children, perhaps because few Ugandan children had IDA or because anemia has a multifactorial etiology that may differ between countries.

We further investigated the effects of a range of iron markers on malaria-specific antibody levels. TSAT, an indicator of low levels of circulating iron, may more accurately reflect what iron status would be in the absence of malaria and inflammation compared to ferritin [33, 34]. TSAT < 10% was associated with reduced anti-AMA-1, although not anti-MSP-1, antibody levels in adjusted models. Hepcidin, the iron regulatory hormone, controls the absorption and recycling of iron, and is regulated by iron stores, infection and erythropoietic drive [35]. We found that increased hepcidin levels were associated with increased anti-MSP-1 antibody levels in overall multivariable analyses. In a previous study of 324 Kenyan children we similarly found that hepcidin levels were positively associated with anti-AMA-1 and anti-MSP-2 antibody levels [21]. In contrast to the other iron markers, we found that increased sTfR levels, an indicator of both increased ID and erythropoietic drive, were associated with increased anti-AMA-1 antibody levels. This might be explained by the strong association between sTfR levels and malaria [36], thus increased sTfR could indicate recent malaria exposure rather than ID.

How might ID lead to reduced malaria-specific antibody levels? One explanation is that iron may play a critical role in humoral immunity and particularly in antigen-specific antibody production as suggested by recent studies [7, 8, 11]. A missense mutation in transferrin receptor 1, necessary for iron uptake by cells, was associated with defective B-cell proliferation and reduced IgG production in children and in mouse models [8]. ID is similarly associated with markedly reduced antigen-specific antibody responses, likely due to impaired iron-dependent histone 3 lysine 9 demethylation, critical for B cell proliferation [7]. There is sparse literature in humans with conflicting findings. ID has been associated with reduced IgG antibodies, including to pneumococcal antigen [8–11], although some studies report little association [14, 16]. ID has also been associated with weakened antibody responses to measles, diphtheria, whooping cough, and tetanus vaccines in some studies [7, 13] but not others [17, 18].

Reduced malaria-specific antibodies in iron deficient children may also be explained by the complex relationship between iron status and malaria. ID has some protective effect against malaria infection in children [37], and thus iron-deficient children may have fewer malaria episodes leading to reduced malariaspecific antibody levels. Another explanation is that malaria influences measures of iron status. Ferritin levels are elevated for a prolonged period after a malaria infection, even after CRP levels have normalized [34], so that children with low ferritin levels may be less likely to have had recent malaria and thus might have reduced antibody levels. Moreover, the malariaspecific antibodies, anti-AMA-1 and anti-MSP-1, are markers of malaria exposure [38, 39], as well as correlates of naturally acquired immunity against clinical malaria [5, 6, 22]. We adjusted for previous malaria exposure in meta-analyses; however, it is likely that not all previous malaria was fully accounted for.

Strengths of our study included its large sample size of 1794 children from cohorts of varying malaria intensity in Kenya and Uganda. We also measured specific malaria antibodies known to contribute to immunity to clinical malaria [6, 22], assayed a wide range of markers of iron status, and adjusted for known potential confounders in our models. There were also some important limitations to our study. First, apart from malaria parasitemia, we did not have standardized measures for malaria exposure available for all cohorts, however, we conducted meta-analyses that accounted for measures of previous malaria, including malaria exposure index, incidence of clinical malaria, and time since a malaria episode, as available for each study site. Another limitation was that ID was defined using WHO guidance [29], which adjusts ferritin levels for inflammation (CRP > 5 mg/ dl), however since ferritin levels are elevated for a prolonged period after CRP levels have normalized following malaria infection [34], lower ferritin levels could also reflect less recent malaria exposure. In addition to adjusting for recent malaria, we also defined ID using TSAT, which is less influenced by inflammation and malaria [33, 34], although this marker was not available for all cohorts. A further limitation of our study is that elevated anti-AMA-1 and anti-MSP-1 antibody levels may not be mechanically related with clinical protection against malaria. However, even as correlates of exposure the responses may still be useful indicators of the host’s immunological response. Antibody levels were also measured using different pools of malaria-hyperimmune control sera in different laboratories in Kenya and Uganda, although protocols were similar between sites. Despite these differences, our findings were notably similar between the different study sites.

In summary, we found that ID was associated with lower levels of anti-AMA-1 and anti-MSP-1 malaria antibodies, known to be important in antibody-mediated immunity to clinical malaria in African children [5, 6, 22]. Our findings are supported by studies demonstrating that iron is critical for the development of humoral immunity [7, 8]. ID is highly prevalent among African children, and it is not known whether improving iron status might improve immune function and reduce disease burden. The current study supports WHO recommendations to offer iron supplementation coupled with malaria treatment in malaria endemic regions to prevent and treat iron deficiency [40]. Further research to infer causality between ID and malaria immunity, such as randomized controlled trials of the effects of iron supplementation on malaria antibody levels are needed, as well as further studies to assess associations between ID and malaria vaccine responses.

## Supplementary Material

Supplementary Figure 1

Supplementary Figure 2

Supplementary Figure 3

Supplementary Material

## Figures and Tables

**Table 1:** Characteristics of Study Participants

					Kenya		Uganda	
**Characteristics**	**Overall n = 1794**		**Junju n = 582**		**RTS,S n = 342**		**EMaBS n = 870**	
Median age months (IQR)^a^	24.0 (18.69, 34.93)		2774 (18.89, 51.74)		12.61 (9.08, 16.79)		24.08 (23.97, 35.90)	
Sex: female no./total (%)	877/1794 (48.89)		284/582 (48.80)		168/342 (49.12)		425/870 (48.85)	
Underweight, no./total (%)^b^	166/1211 (13.71)		88/344 (25.58)		na		78/867 (9.00)	
Inflammation, no./total (%)^c^	478/1749 (2784)		180/564 (31.91)		95/339 (28.02)		212/846 (25.06)	
Malaria parasitemia, no./total (%)^d^	262/1588 (16.50)		202/582 (34.70)		3/152 (1.97)		57/854 (6.67)	
Malaria exposure index, median (IQR)^e^	0.32 (1.5^-11^, 0.63)		0.50 (6.16^-11^, 0.75)		0.09 (2.17^-14^, 0.32)		na	
Malaria incidence, gmean (95% CI)^f^	1.02 (.93, 1.10)		1.67 (1.41, 1.96)		na		.92 (.84, 1.01)	
ID, low ferritin, no./total (%)^g^	528/1683 (31.37)		111/552 (20.11)		153/335 (45.67)		264/796 (33.17)	
ID, low TSAT, no./total (%)^h^	425/889 (47.81)		220/554 (39.71)		205/335 (61.19)		n/a	
IDA, no./total (%)^i^	171/1265 (13.52)		90/488 (18.44)		n/a		80/762 (10.05)	
Anemia, no./total (%)^j^	585/1367 (42.79)		359/516 (69.57)		n/a		220/834 (26.38)	
**Biomarkers and malaria antibodies**	**n**	**gmean (95% CI)**	**n**	**gmean (95% CI)**	**n**	**gmean (95% CI)**	**n**	**gmean (95% CI)**
Ferritin (μg/L)	1683	23.44 (22.29, 24.64)	552	32.68 (30.0, 35 .61)	335	1740 (15.70, 19.30)	796	21.10 (19.61,22.69)
TSAT (%)	889	10.76 (10.34, 11.18)	554	11.81 (11.20, 12.42)	335	9.21 (8.69, 9.77)		n/a
Hepcidin (μg/L)	1694	6.71 (6.34, 7.10)	546	7.01 (6.4, 7.72)	298	5.69 (4.92,6.58)	850	6.92 (6.39, 7.48)
Iron (μg/dL)	900	744 (719, 769)	561	7.93 (7.62, 8.31)	298	6.68 (6.34, 703)		n/a
Transferrin (mg/dL)	1749	2.70 (2.68, 2.73)	568	2.70 (2.62, 2.72)	337	2.87 (2.81,2.92)	844	2.68 (2.64, 2.72)
sTfR (mg/L)	1765	11.07 (10.68, 11.48)	573	18.22 (1762, 18.91)	339	18.04 (17.35,18.76)	853	6.53 (6.23, 6.85)
Hemoglobin (g/dL)	1367	10.85 (10.76, 10.94)	516	10.15 (10.02, 10.31)		n/a	834	11.30 (11.2, 11.40)
CRP (mg/L)	1749	1.77 (1.64, 1.91)	564	2.31 (2.03, 2.61)	339	2.00 (.56, .79)	846	1.41 (1.26, 1.58)
AMA-1 (AU/mL)	1678	55.84 (50.76, 61.42)	582	196.99 (164.95, 235.25)	342	38.06 (35.74,40.51)	754	25.11 (22.15, 28.47)
MSP-1 (AU/mL)	1765	181.60 (170.0, 193.99)	582	354.04 (313.29, 400.08)	342	14713 (132.29, 163.64)	841	124.63 (113.92, 136.36)

Abbreviations: AMA-1, apical merozoite antigen 1; CI, confidence interval; CRP, C-reactive protein; gmean, geometric mean; IDA, iron deficiency anemia; IQR, interquartile range; MSP-1, merozoite surface protein 1; MUAC, mid-upper arm circumference; n/a, not available; sTfR, soluble transferrin receptor; TSAT, transferrin saturation.

a Median age at time of iron measurement.

b Underweight was defined as weight for age *z*-score < -2.

c inflammation, C-reactive protein > 5 mg/L.

d Malaria parasitemia, *Plasmodium falciparum* parasitemia at any density at the time of iron measurement.

e Malaria exposure index, a marker of the level of a child’s exposure to malaria and was calculated as the distance-weighted prevalence of clinical malaria within 1 km radius of the child’s residence.

f Malaria incidence, total number of malaria episodes before the time of iron measurement/follow-up time.

g Iron deficiency as low ferritin, plasma ferritin < 12 μg/L or < 30 μg/L in the presence of inflammation in children < 5 years or < 15 μg/L in children ≥ 5 years or as

h low TSAT, TSAT < 10%.

i IDA as ID defined by low ferritin and anemia.

j Anemia as hemoglobin < 11 g/dL in children aged 0 to 4 years or hemoglobin < 11.5 g/dL in children above 4 years.

**Table 2:** Association Between Iron Deficiency and AMA-1 and MSP-1 Antibody Levels in Univariable and Multivariable Regression Models

Cohort	Iron Replete		Iron Deficient		Unadjusted		Adjusted	
	n	Geometric Mean (95% CI)	n	Geometric Mean (95% CI)	Coefficient (95% CI)	*P*-value	Coefficient (95% CI)	*P*-value
**Log AMA-1 antibody**								
**ID, low ferritin**								
Overall n = 1583	1094	7727 (68.74, 8761)	612	26.91 (23.77, 30.47)	−1.05 (−1.26, −.85)	<.0001	−.46 (−.66, −.25)	<.0001
Junju n = 552	441	284.53 (232.90, 34761)	111	40.77 (30.04, 55.32)	−1.94 (−2.37, −1.51)	<.0001	−.51 (−.89, −.13)	. 01
EMaBS n = 696	471	29.49 (25.02, 34.76)	225	1771 (14.42, 21.75)	−.51 (−.79, −.23)	<.0001	−.36 (−.64, − .08)	. 01
RTSS n = 335	182	39.71 (36.22, 43.53)	153	36.87 (33.90, 40.10)	−.07 (−.20, .05)	.24	−.03 (−.17, .10)	.64
**ID, low TSAT**								
Overall n = 887	464	176.19 (145.91,212.76)	425	59.64 (51.20, 69.47)	−1.08 (−1.32, −.83)	<.0001	−.34 (−.59, −.10)	.007
Junju n = 552	334	318.79 (253.45, 400.97)	220	92.15 (70.04, 121.26)	−1.24 (−1.60, −.88)	<.0001	−.30 (−.61, .01)	.06
RTSS n = 335	130	38.40 (34.58, 42.66)	205	3739 (34.59, 40.41)	−.02 (−.15, .10)	.68	.05 (−.09, .18)	.49
**Log MSP-1 antibody**								
**ID, low ferritin**								
Overall n = 1655	1137	213.51 (196.33, 232.19)	518	126.21 (113.14, 140.80)	−.53 (−.67, −.38)	<.0001	−.33 (−.50, −.17)	<.0001
Junju n = 552	441	418.38 (364.24, 480.57)	111	166.45 (128.78, 215.12)	−.92 (−1.22, −.82)	<.0001	−.50 (−.83, −.15)	.005
EMaBS n = 768	514	113.98 (119.57, 150.12)	254	108.43 (90.99, 126.85)	−.22 (−.42, −.02)	.03	−.20 (−.40, .01)	.07
RTSS n = 335	182	155.98 (134.65, 180.70)	153	134.93 (115.01, 158.30)	−.15 (−.39, .09)	.23	−.06 (−.27, .15)	.60
**ID, low TSAT**								
Overall n = 889	464	305.18 (268.13, 34735)	425	208.09 (182.78, 236.91)	−.38 (−.57, −.20)	<.0001	−.02 (−.24, .20)	.83
Junju n = 554	334	405.29 (345.72, 475.11)	220	28797 (234.89, 353.05)	−.50 (−.74, −.26)	<.0001	−.02 (−.28, .25)	.86
RTSS n = 335	130	14723 (125.45, 172.79)	205	146.84 (12720, 169.51)	−.003 (−.25, .24)	.98	.05 (−.16, .26)	.65

Overall models, including all cohorts, were adjusted for age, sex, inflammation, study site, and malaria parasitemia at time of antibody measurement. For individual cohorts we further adjusted for malaria exposure index (in Kenyan cohorts), for malaria vaccination (RTS,S cohort), and for malaria incidence, time since last malaria episode, and time between iron and antibody measurement (EMaBS cohort). Iron deficiency was defined as (a) ID, low ferritin; plasma ferritin < 12 μg/L or < 30 μg/L in the presence of inflammation in children < 5 years or < 15 μg/L in children ≥ 5 years and (b) ID, low TSAT (TSAT < 10%).

Abbreviations: EMaBS, Entebbe Mother and Baby Study; ID; iron deficiency, TSAT; transferrin saturation.
