# Supplementary figures and images for: Iron Deficiency Is Associated With Reduced Levels of Plasmodium falciparum-specific Antibodies in African Children
Source: Clin Infect Dis. Author manuscript; Available in PMC 2021 Jul 8. (PMC8246895; doi:10.1093/cid/ciaa728)

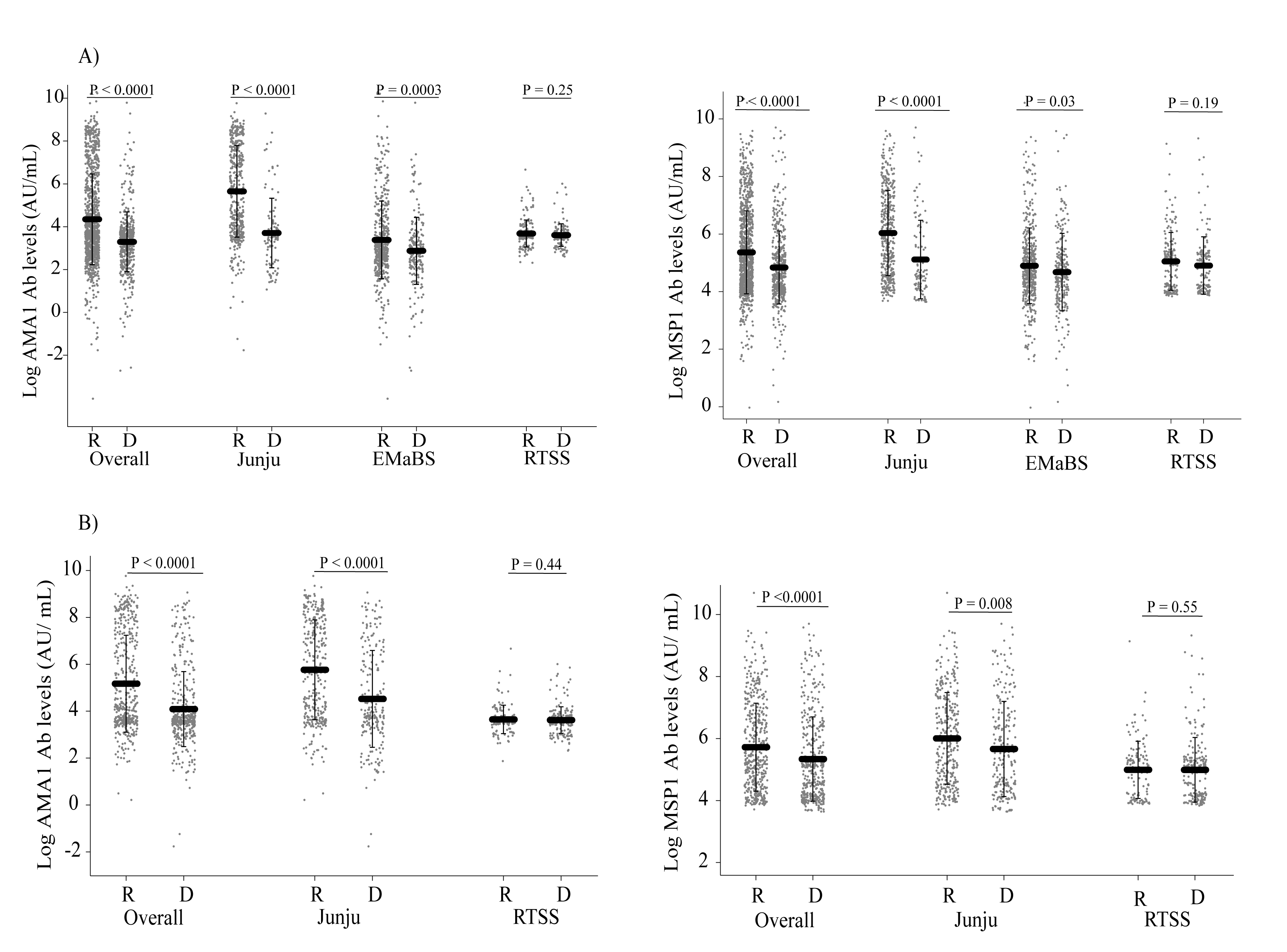

Supplement: Supplementary Figure 1 [file EMS127233-supplement-Supplementary_Figure_1.png]

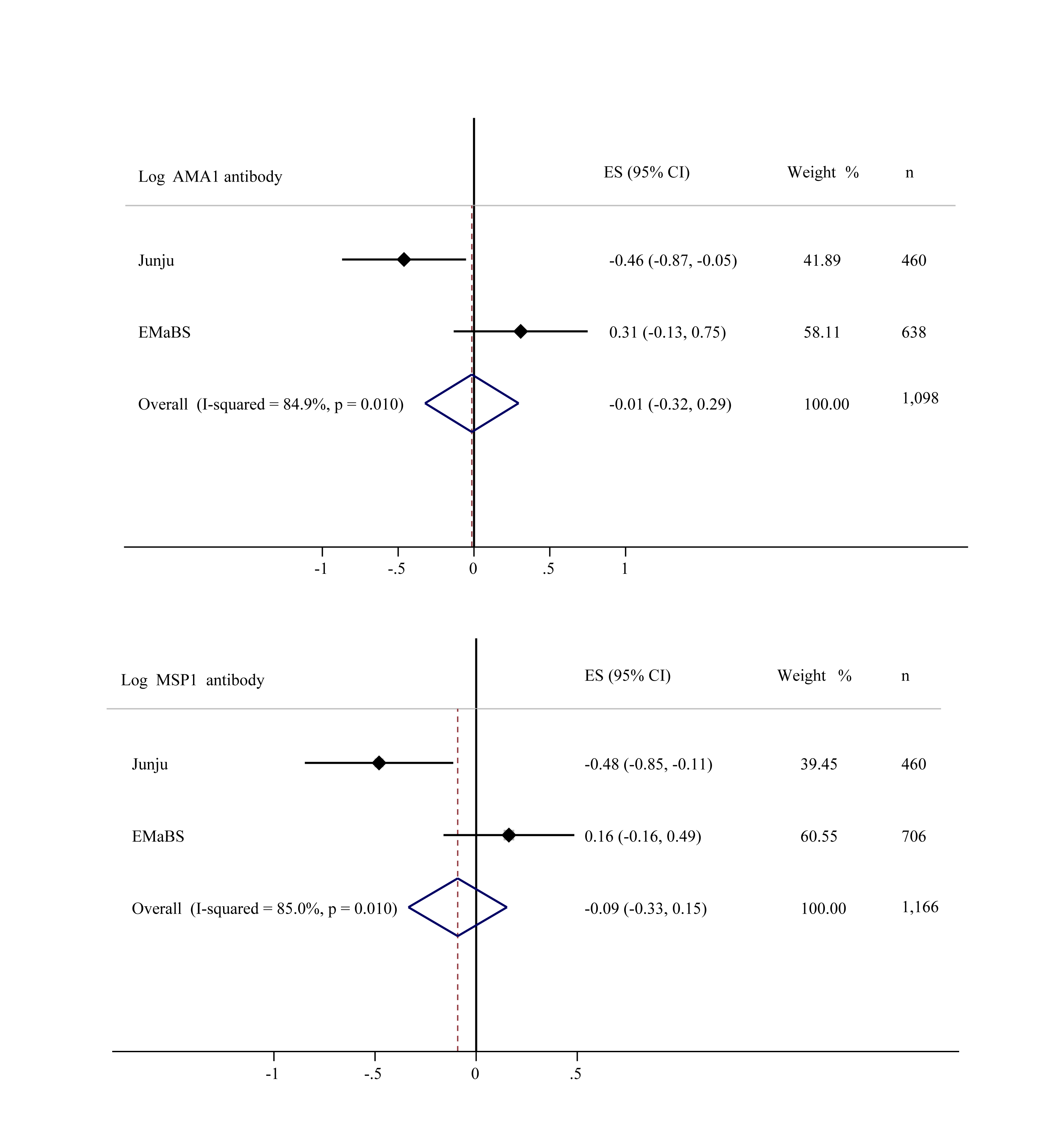

Supplement: Supplementary Figure 2 [file EMS127233-supplement-Supplementary_Figure_2.png]

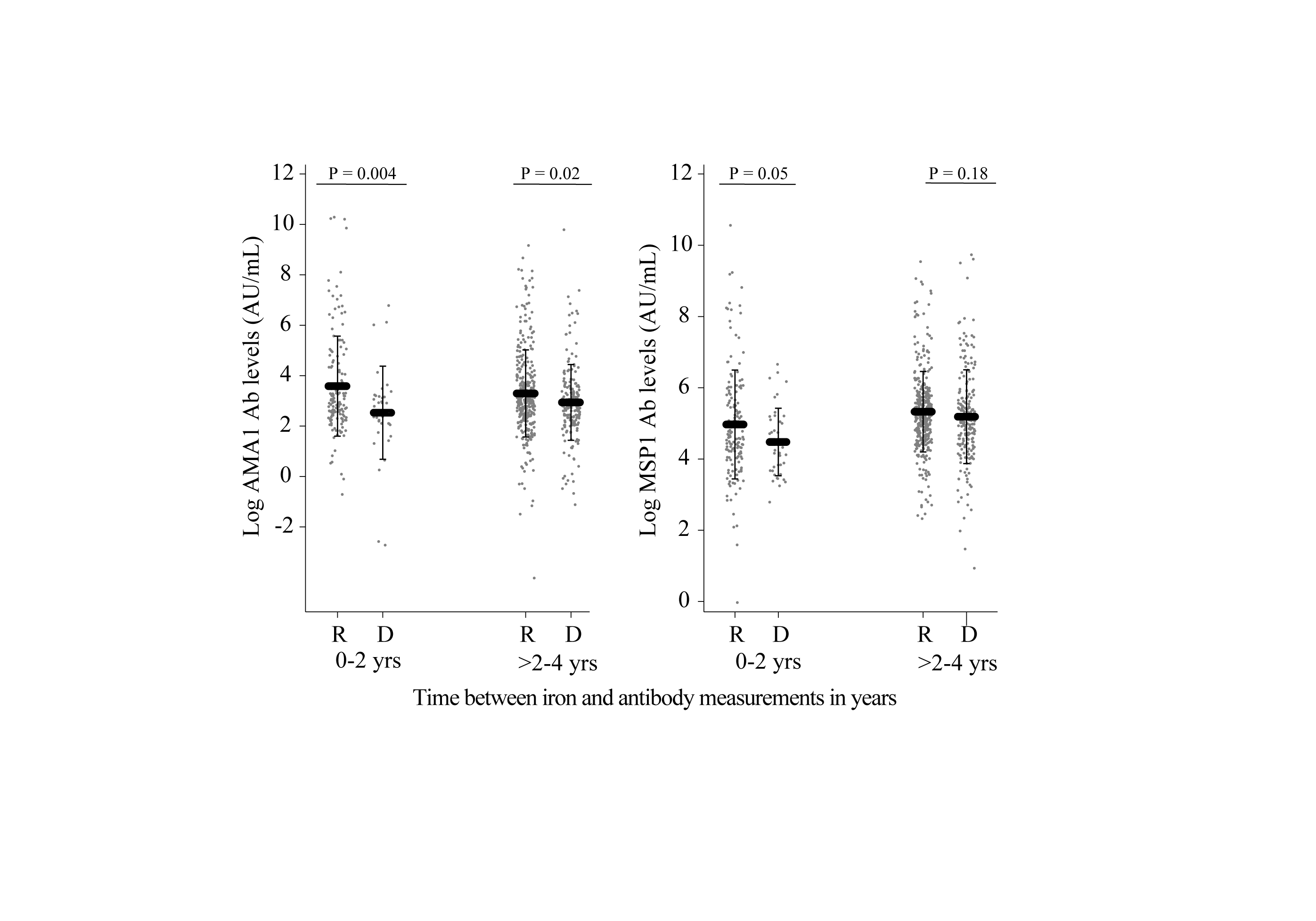

Supplement: Supplementary Figure 3 [file EMS127233-supplement-Supplementary_Figure_3.png]
