# Supplementary material for: Iron Deficiency Is Associated With Reduced Levels of Plasmodium falciparum-specific Antibodies in African Children
Source: Clin Infect Dis. Author manuscript; Available in PMC 2021 Jul 8. (PMC8246895; doi:10.1093/cid/ciaa728)
Supplement: Supplementary Material [file EMS127233-supplement-Supplementary_Material.docx]

S**upplementary Material**

Supplementary Table 1. Factors associated with malaria antibody levels in univariable regression analyses

| **Malaria antibody** | **Variables** | **n** | **Coefficient (95% CI)** | **P value** |
| --- | --- | --- | --- | --- |
| **Log AMA1 antibody** | | | | |
| Overall | Gender: females | 1,678 | -0.002 (-0.01, 0.01) | 0.97 |
|  | Age in months | 1,678 | 3.31 (2.96, 3.66) | <0.0001 |
|  | ^a^Underweight | 1,095 | 0.03 (0.02, 0.04) | <0.0001 |
|  | ^b^Inflammation | 1,637 | 0.04 (0.03, 0.05) | <0.0001 |
|  | ^c^Malaria parasitemia | 1,475 | 0.09 (0.08, 0.09) | <0.0001 |
|  | ^d^Malaria exp index | 833 | -0.007 (-0.02, 0.01) | 0.27 |
|  | ^e^Malaria incidence | 946 | 0.89 (-4.94, 6.72) | 0.76 |
|  | Study site |  |  |  |
|  | EMaBS (reference) | 870 | Reference | - |
|  | Junju | 582 | 2.05 (1.86,2.25) | <0.0001 |
|  | RTSS | 342 | 0.37 (0.09, 0.63) | 0.01 |
| **Log MSP1 antibody** | | | | |
| Overall | Gender: females | 1765 | 0.001 (-0.02, 0.02) | 0.91 |
|  | Age in months | 1765 | 2.77 (2.27, 3.27) | <0.0001 |
|  | ^a^Underweight | 1,182 | 0.01 (-0.004, 0.02) | 0.17 |
|  | ^b^Inflammation | 1721 | 0.02 (0.001,0.03) | 0.03 |
|  | ^c^Malaria parasitemia | 1560 | 0.06 (0.05, 0.08) | <0.0001 |
|  | ^d^Malaria exp index | 833 | 0.02 (0.01,0.04) | 0.01 |
|  | ^e^Malaria incidence | 817 | 0.30 (-8.48, 9.07) | 0.94 |
|  | Study site |  |  |  |
|  | EMaBS (Reference) | 870 | Reference | - |
|  | Junju | 582 | 1.04 (0.90, 1.18) | <0.0001 |
|  | RTSS | 342 | 0.16 (0.90, 1.18) | 0.05 |

Malaria exp index, median malaria exposure index.  ^a^Underweight was defined as weight for age z-score < -2 (not available for Ngerenya and RTSS). ^b^Inflammation, C-reactive protein > 5mg/L. ^c^Malaria parasitemia, *Plasmodium falciparum* parasitaemia at any density at the time of antibody measurement. ^d^Malaria exposure index, a marker of the level of a child’s exposure to malaria and was calculated as the distance-weighted prevalence of clinical malaria within 1 km radius of the child’s residence (not available for Uganda). ^e^Malaria incidence, total number of malaria events before time of iron measurement/follow up time (not available for Ngerenya and RTSS).

Supplementary Table 2: Regression models showing association between iron deficiency and AMA1 and MSP1 antibody levels in univariable models and multivariable models additionally adjusted for underweight (adjustment 2)

|  |  | **Iron replete** |  | **Iron deficient** | **Unadjusted** |  | **Adjustment 1** |  | **Adjustment 2** |  |
| --- | --- | --- | --- | --- | --- | --- | --- | --- | --- | --- |
| **Cohort** | **n** | **Geometric mean**  **(95% CI)** | **n** | **Geometric mean**  **(95% CI)** | **Coefficient**  **(95% CI)** | **P value** | **Coefficient**  **(95% CI)** | **P value** | **Coefficient**  **(95% CI)** | **P value** |
| **Log AMA1 antibody** |  |  |  |  |  |  |  |  |  |  |
| **ID, low ferritin** |  |  |  |  |  |  |  |  |  |  |
| Overall n=1,583 | 1,094 | 77.27 (68.15, 87.61) | 612 | 26.91 (23.77, 30.47) | -1.05 (-1.26, -0.85) | <0.0001 | -0.46 (-0.66, -0.25) | <0.0001 | -0.42 (-0.66, -0.19) | <0.0001 |
| Junju n=552 | 441 | 284.53 (232.90, 347.61) | 111 | 40.77 (30.04, 55.32) | -1.94 (-2.37, -1.51) | <0.0001 | -0.51 (-0.89, -0.13) | 0.01 | -0.42 (-0.87, 0.02) | 0.06 |
| EMaBS n=696 | 471 | 29.49 (25.02, 34.76) | 225 | 17.71 (14.42, 21.75) | -0.51 (-0.79, -0.23) | <0.0001 | -0.36 (-0.64, -0.08) | 0.01 | -0.36 (-0.64, -0.08) | 0.01 |
| RTSS n=335 | 182 | 39.71 (36.22, 43.53) | 153 | 36.87 (33.90, 40.10) | -0.07 (-0.20, 0.05) | 0.24 | -0.03 (-0.17, 0.10) | 0.64 | na | na |
| **ID, low TSAT** |  |  |  |  |  |  |  |  |  |  |
| Overall n=889 | 464 | 176.19 (145.91, 212.76) | 425 | 59.64 (51.20, 69.47) | -1.08 (-1.32, -0.83) | <0.0001 | -0.34 (-0.59, -0.10) | 0.007 | na | na |
| Junju n=552 | 334 | 318.79 (253.45, 400.97) | 220 | 92.15 (70.04, 121.26) | -0.84 (-1.09, -0.59) | <0.001 | -0.30 ( -0.61, 0.01) | 0.06 | -0.32 (-0.68,0.04) | 0.09 |
| RTSS n=335 | 130 | 38.40 (34.58, 42.66) | 205 | 37.39 (34.59, 40.41) | -0.07 (-0.23, 0.09) | 0.39 | 0.05 (-0.09, 0.18) | 0.49 | na | na |
| **IDA** |  | **No IDA** |  | **IDA** |  |  |  |  |  |  |
| Overall n=1244 | 1,011 | 62.26 (54.19, 71.53) | 159 | 33.67 (26.56, 42.67) | -0.61 (-0.98, -0.25) | 0.001 | -0.07 (-0.37, 0.23) | 0.64 | 0.01 (-0.31, 0.33) | 0.94 |
| Junju n=448 | 398 | 249.57 (201.14, 309.67) | 90 | 37.46 (27.08, 51.90) | -1.49 (-1.81, -1.15) | <0.0001 | -0.46 (-0.87, -0.05) | 0.03 | -0.19 (-0.63, 0.25) | 0.40 |
| EMaBS n=667 | 599 | 25.03 (21.66, 28.91) | 68 | 29.18 (20.44, 41.67) | 0.15 (-0.29, 0.60) | 0.50 | 0.32 (-0.13, 0.76) | 0.17 | 0.30 (-0.14, 0.75) | 0.18 |
| **Log MSP1 antibody** |  |  |  |  |  |  |  |  |  |  |
|  |  | **Iron replete** |  | **Iron deficient** |  |  |  |  |  |  |
| **ID, low ferritin** |  |  |  |  |  |  |  |  |  |  |
| Overall n=1655 | 1137 | 213.51 (196.33, 232.19) | 518 | 126.21 (113.14, 140.80) | -0.53 (-0.67, -0.38) | <0.0001 | -0.33 (-0.50, -0.17) | <0.0001 | -0.30(-0.49, -0.12 | 0.001 |
| Junju n=552 | 441 | 418.38 (364.24, 480.57) | 111 | 166.45 (128.78, 215.12) | -0.92 (-1.22, -0.62) | <0.0001 | -0.50 (-0.83, -0.18) | 0.003 | -0.51 (-0.91, -0.12) | 0.01 |
| EMaBS n=768 | 514 | 113.98 (119.57, 150.12) | 254 | 108.43 (90.99, 126.85) | -0.22 (-0.42, -0.02) | 0.03 | -0.20 (-0.40, 0.01) | 0.07 | -0.19 (-0.40, 0.02) | 0.07 |
| RTSS n=335 | 182 | 155.98 (134.65, 180.70) | 153 | 134.93 (115.01, 158.30) | -0.15 (-0.39, 0.09) | 0.23 | -0.06 (-0.27, 0.15) | 0.60 | na | na |
| **ID, low TSAT** |  |  |  |  |  |  |  |  |  |  |
| Overall n=889 | 464 | 305.18 (268.13, 347.35) | 425 | 208.09 (182.78, 236.91) | -0.38 (-0.57, -0.20) | <0.0001 | -0.02 (-024, 0.20) | 0.83 | na | na |
| Junju n=554 | 334 | 405.29 (345.72, 475.11) | 220 | 287.97 (234.89, 353.05) | -0.50 (-0.74, -0.26) | <0.0001 | -0.02 (-0.30, 0.25) | 0.86 | 0.02 (-0.34,0.38) | 0.92 |
| RTSS n=335 | 130 | 147.23 (125.45, 172.79) | 205 | 146.84 (127.20, 169.51) | -0.003 (-0.25, 0.24) | 0.98 | 0.05 (-0.16, 0.26) | 0.65 | na | na |
| **IDA** |  | **No IDA** |  | **IDA** |  |  |  |  |  |  |
| Overall n=1238 | 1068 | 194.86 (177.99, 213.32) | 170 | 151.38 (122.79, 186.63) | -0.25 (-0.49, -0.01) | 0.04 | -0.15 (-0.39, 0.09) | 0.21 | -0.15 (-0.39, 0.09) | 0.21 |
| Junju n=488 | 412 | 404.71 (350.09, 467.86) | 91 | 161.21 (120.84, 215.07) | -1.12 (-1.41, -0.82) | <0.0001 | -0.48 (-0.84, -0.11) | 0.01 | -0.48 (-0.92, -0.05) | 0.03 |
| EMaBS n=735 | 656 | 123.13 (111.25, 136.26) | 79 | 140.79 (103.21 (192.05) | 0.13 (-1.8, 0.44) | 0.40 | 0.16 (-0.16, 0.49) | 0.33 | 0.22 (-0.10, 0.55) | 0.17 |

ID; Iron deficiency, IDA; Iron deficiency anemia, TSAT; Transferrin saturation. Overall models including all cohorts were adjusted for age, gender, inflammation, study site and malaria parasitemia at time of antibody measurement (in adjustment 1). For individual cohorts we further adjusted for malaria exposure index (in Kenyan cohorts), malaria vaccination (RTS,S cohort) and malaria incidence, time since last malaria episode and time between iron and antibody measurement in the Ugandan (EMaBS) cohort. In adjustment 2, we additionally adjusted for underweight (available for Junju and EMaBS cohorts only). Iron deficiency was defined as a) ID, low ferritin; plasma ferritin < 12µg/L or < 30µg/L in the presence of inflammation in children < 5 years or < 15µg/L in children ≥ 5 years and b) ID, low TSAT (TSAT <10%).

Supplementary Table 3. Univariable and multivariable linear regression of individual iron biomarkers on AMA1 and MSP1 antibody levels

|  | **Unadjusted** | | | **Adjusted** | |
| --- | --- | --- | --- | --- | --- |
| **Cohort** | **n** | **Coefficient**  **(95% CI)** | **P value** | **Coefficient**  **(95% CI)** | **P value** |
| **Log AMA1 antibody** | | | | | |
| **Log ferritin** |  |  |  |  |  |
| Overall | 1,583 | 0.64 (0.55, 0.73) | <0.0001 | 0.27 (0.18, 0.37) | <0.0001 |
| Junju | 552 | 0.71 (0.60, 0.82) | <0.0001 | 0.24 (0.06, 0.41) | 0.01 |
| RTSS | 335 | 0.03 (-0.06, 0.11) | 0.52 | -0.04 (-0.11, 0.03) | 0.30 |
| EMaBS | 696 | 0.35 (0.22, 0.47) | <0.0001 | 0.24 (0.10, 0.38) | 0.001 |
| **Log TSAT** |  |  |  |  |  |
| Overall | 889 | 0.95 (0.75, 1.16) | <0.0001 | 0.26 (0.05, 0.47) | 0.01 |
| Junju | 554 | 0.81 (0.61, 1.01) | <0.0001 | 0.19 (-0.06, 0.45) | 0.14 |
| RTSS | 334 | 0.05 (-0.10, 0.19) | 0.52 | -0.07 (-0.20, 0.05) | 0.24 |
| **Log hepcidin** |  |  |  |  |  |
| Overall | 1,580 | 0.22 (0.14, 0.30) | <0.0001 | 0.01 (-0.06, 0.09) | 0.77 |
| Junju | 546 | 0.39 (0.28, 0.49) | <0.0001 | 0.12 (-0.02, 0.26) | 0.09 |
|  |  |  |  |  |  |
| RTSS | 298 | -0.03 (-0.10, 0.04) | 0.36 | -0.05 (-0.11, 0.01) | 0.11 |
| EMaBS | 736 | 0.09 (-0.02, 0.19) | 0.12 | 0.03 (-0.08, 0.14) | 0.59 |
| **Log iron** |  |  |  |  |  |
| Overall | 900 | 0.79 (0.55, 1.03) | <0.0001 | 0.20 (-0.04, 0.42) | 0.10 |
| Junju | 561 | 0.81 (0.47, 1.15) | <0.0001 | 0.08 (-0.20, 0.37) | 0.56 |
| RTSS | 339 | 0.04 (-0.09, 0.17) | 0.53 | -0.02 (-0.17, 0.12) | 0.75 |
| **Transferrin** |  |  |  |  |  |
| Overall | 1,637 | -0.59 (-0.76, -0.42) | <0.0001 | -0.16 (-0.32, 0.001) | 0.05 |
| Junju | 568 | -1.27 (-1.56, --0.97) | <0.0001 | -0.32 (-0.59, -0.05) | 0.02 |
| RTSS | 337 | 0.0.08 (-0.04, 0.20) | 0.17 | 0.12 (-0.003, 0.25) | 0.06 |
| EMaBS | 732 | -0.19 (-0.40, 0.03) | 0.09 | -0.03 (-0.26, 0.18) | 0.75 |
| **Log sTfR** |  |  |  |  |  |
| Overall | 1,652 | 0.69 (0.57, 0.82) | <0.0001 | 0.29 (0.15, 0.44) | <0.0001 |
| Junju | 573 | -0.22 (-0.52, 0.07) | 0.13 | -0.05 (-0.39, 0.30) | 0.79 |
| RTSS | 339 | 0.22 (0.01, 0.44) | 0.05 | 0.29 (0.10, 0.47) | 0.002 |
| EMaBS | 740 | 0.17 (-0.01, 0.34) | 0.08 | 0.08 (-0.10, 0.26) | 0.40 |
| **Hemoglobin** |  |  |  |  |  |
| Overall | 1257 | -0.18 (-0.26, -0.11) | <0.0001 | -0.07 (-0.13, -0.05) | 0.07 |
| Junju | 516 | 0.27 (0.14, 0.38) | <0.0001 | 0.04 (-0.06, 0.14) | 0.47 |
| EMaBS | 724 | -0.16 (-0.26, -0.07) | <0.001 | -0.14 (-0.23, -0.04) | 0.01 |
| **Log MSP1 antibody** | | | | | |
| **Log ferritin** |  |  |  |  |  |
| Overall | 1,655 | 0.26 (0.19, 0.32) | <0.0001 | 0.14 (0.06, 0.22) | 0.001 |
| Junju | 552 | 0.44 (0.33, 0.55) | <0.0001 | 0.17 (0.01, 0.32) | 0.03 |
| RTSS | 335 | 0.12 (-0.01, 0.24) | 0.07 | 0.03 (-0.08, 0.15) | 0.60 |
| EMaBS | 768 | 0.08 (-0.01, 0.17) | 0.09 | 0.07 (-0.04) 0.18 | 0.21 |
| **Log TSAT** |  |  |  |  |  |
| Overall | 889 | 0.40 (0.24, 0.55) | <0.0001 | 0.06 (-0.12, 0.25) | 0.51 |
| Junju | 554 | 0.52 (0.33, 0.71) | <0.0001 | 0.06 (-0.17, 0.29) | 0.61 |
| RTSS | 335 | 0.03 (-0.19, 0.25) | 0.78 | -0.03 (-0.22, 0.16) | 0.75 |
| **Log hepcidin** |  |  |  |  |  |
| Overall | 1,666 | 0.13 (0.06, 0.19) | <0.0001 | 0.07 (0.004, 0.13) | 0.04 |
| Junju | 546 | 0.28 (0.18, 0.38) | <0.0001 | 0.14 (0.02, 0.26) | 0.03 |
| RTSS | 298 | 0.08 (-0.02, 0.17) | 0.13 | -0.004 (-0.09, 0.08) | 0.93 |
| EMaBS | 822 | 0.07 (-0.01, 0.14) | 0.09 | 0.06 (-0.02, 0.14) | 0.16 |
| **Log iron** |  |  |  |  |  |
| Overall | 900 | 0.35 (0.18, 0.53) | <0.0001 | 0.04 (-0.16, 0.25) | 0.69 |
| Junju | 561 | 0.31 (0.07, 0.54) | 0.01 | 0.04 (-0.22, 0.30) | 0.78 |
| RTSS | 339 | 0.07 (-0.16, 0.29) | 0.56 | 0.02 (-0.20, 0.24) | 0.84 |
| **Transferrin** |  |  |  |  |  |
| Overall | 1,720 | -0.20 (-0.32, -0.08) | 0.001 | -0.08 (-0.21, 0.04) | 0.23 |
| Junju | 568 | -040 (-0.62, -0.19) | <0.0001 | -0.12 (-0.36, 0.13) | 0.34 |
| RTSS | 337 | -0.02 (-0.22, 0.20) | 086 | 0.06 (-0.14, 0.26) | 0.53 |
| EMaBS | 815 | -0.04 (-0.20, 0.12) | 0.61 | -0.01 (-0.18, 0.16) | 0.88 |
| **Log sTfR** |  |  |  |  |  |
| Overall | 1,736 | 0.29 (0.21, 0.38) | <0.0001 | 0.08 (-0.03, 0.19) | 0.15 |
| Junju | 573 | -0.04 (-0.31, 0.23) | 0.76 | -0.11 (-0.42, 0.21) | 0.50 |
| RTSS | 339 | 0.13 (-0.20, 0.45) | 0.44 | 0.31 (0.02, 0.59) | 0.03 |
| EMaBS | 824 | -0.03 (0.16, 0.09) | 0.56 | -0.06 (-0.19, 0.07) | 0.37 |
| **Haemoglobin** |  |  |  |  |  |
| Overall | 1,339 | -0.06 (-0.11, -0.01) | 0.02 | 0.02 (-0.03, 0.08) | 0.53 |
| Junju | 516 | 1.14 (0.43, 1.87) | 0.002 | 0.05 (-0.04, 0.14) | 0.32 |
| EMaBS | 806 | 0.01 (-0.05, 0.08) | 0.71 | 0.03 (-0.04, 0.10) | 0.42 |

Log TSAT, log transferrin saturation; log sTfR, log soluble transferrin receptor; MSP1, merozoite surface protein1; AMA1, apical merozoite protein 1. Not all iron markers were available for all cohorts. Overall models including all cohorts were adjusted for age, gender, inflammation, study site and malaria parasitemia at time of antibody measurement. For individual cohorts we further adjusted for malaria exposure index (in Kenyan cohorts), malaria vaccination (RTS,S cohort) and malaria incidence, time since last malaria episode and time between iron and antibody measurement in the Ugandan (EMaBS) cohort.

**Supplementary Figure 1**: Scatter plots showing the mean and confidence intervals for AMA1 and MSP1 antibody levels by iron status; A) ID, low ferritin and B) ID, low TSAT. R, iron-replete; D, iron-deficient; TSAT, transferrin saturation; MSP1, merozoite surface protein 1; AMA1, apical merozoite antigen 1. Iron deficiency was defined as a) ID, low ferritin; plasma ferritin < 12µg/L or < 30µg/L in the presence of inflammation in children < 5 years or < 15µg/L in children ≥ 5 years and b) ID, low TSAT (TSAT <10%). P values calculated using Student’s T test.

**Supplementary Figure 2**: Meta-analyses of association of iron deficiency anemia with AMA1 and MSP1 malaria antibodies. Regression models were adjusted for age, gender, inflammation and malaria parasitemia in all individual cohorts with additional adjustment for malaria exposure index in Kenyan cohorts, malaria vaccination (RTS,S cohort) and malaria incidence, time since last malaria episode, and time between iron and antibody measurements in the Ugandan (EMaBS) cohort. Iron deficiency anemia was defined as iron deficiency and hemoglobin < 11g/dL in children aged 0 to 4 years or hemoglobin < 11.5 g/dL in children above 4 years. Malaria exposure index, a marker of the level of a child’s exposure to malaria was calculated as the distance-weighted prevalence of clinical malaria within 1 km radius of the child’s residence. Prior malaria incidence was defined as total number of malaria episodes before the time of iron measurement / follow up time. MSP1, merozoite surface protein1; AMA1, apical merozoite protein 1; ES, effect size.

**Supplementary Figure 3**: Scatter plots showing the mean and confidence interval of AMA1 and MSP1 antibody levels by iron status. Children were stratified according to the time between iron status and antibody measurements. R, iron replete; D, iron deficient; AMA1, merozoite surface protein 1; AMA1, apical merozoite protein1; ID, iron deficiency. Iron deficiency was defined as plasma ferritin < 12µg/L or < 30µg/L in the presence of inflammation in children < 5 years or < 15µg/L in children ≥ 5 years. In the EMaBS cohort malaria antibodies were measured in all children at 5 years and iron status was measured at earlier timepoints.
